# Supplementary material for: Seminoma and Embryonal Carcinoma Footprints Identified by Analysis of Integrated Genome-Wide Epigenetic and Expression Profiles of Germ Cell Cancer Cell Lines
Source: PLoS One. 2014 Jun 2;9(6):e98330. doi: 10.1371/journal.pone.0098330 (PMC4041891; doi:10.1371/journal.pone.0098330)
Supplement: File S1 — ZIP file containing DMRforPairs output for significant regions. Please start from the html files. (ZIP) [file pone.0098330.s008.zip › figures/41143.pdf]

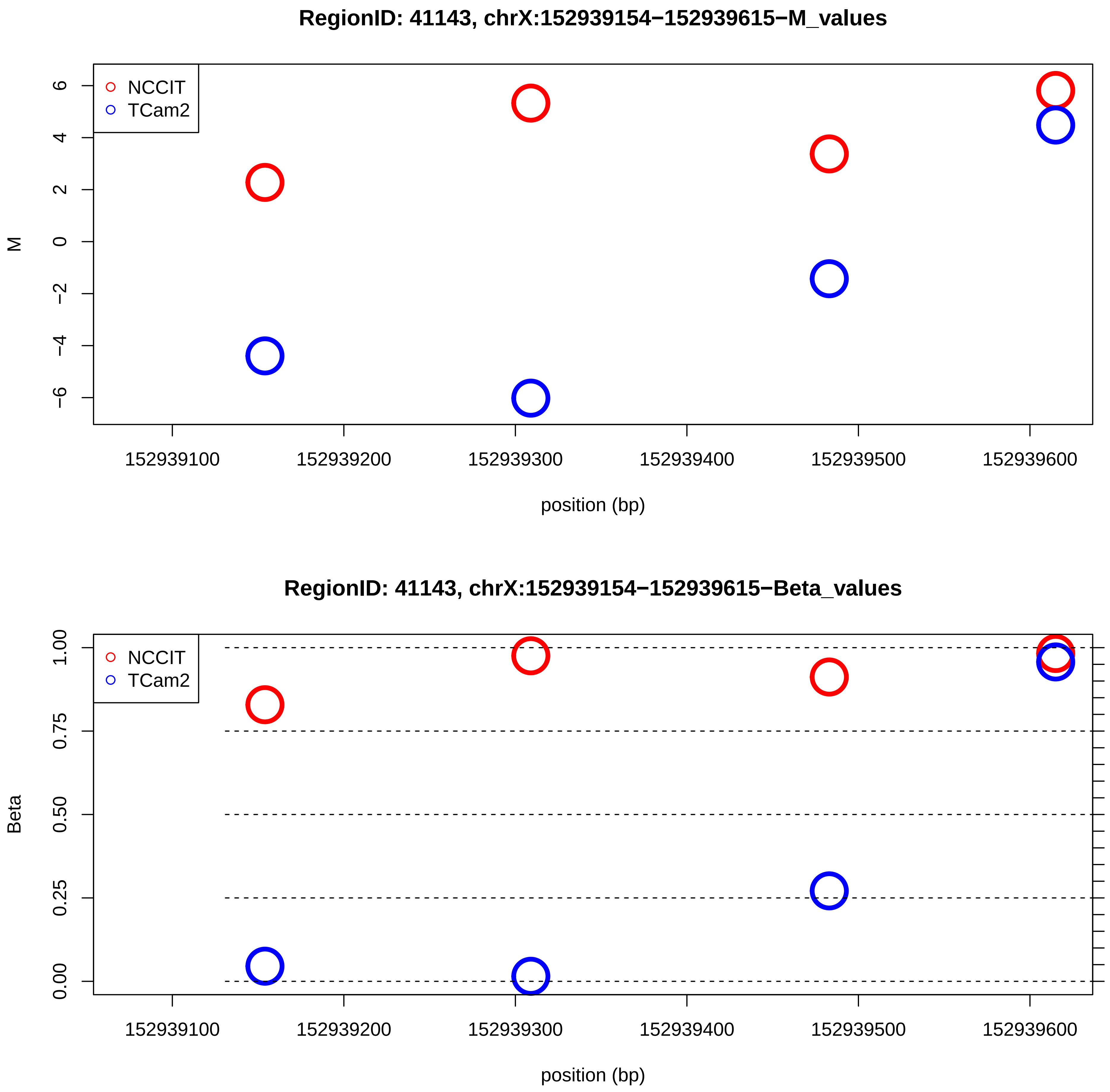

RegionID: 41143, chrX:152939154−152939615−M_values
NCCIT
TCam2
M
6
4
2
0
−2
−4
−6
152939100
152939200
152939300
152939400
152939500
152939600
position (bp)
RegionID: 41143, chrX:152939154−152939615−Beta_values
NCCIT
TCam2
Beta
1.00
0.75
0.50
0.25
0.00
152939100
152939200
152939300
152939400
152939500
152939600
position (bp)
